# Evaluation of the Effectiveness of Various Autophagy Inhibitors in A549 Cancer Stem Cells

**DOI:** 10.32607/actanaturae.11891

**Published:** 2023

**Authors:** K. V. Aleksandrova, I. I. Suvorova

**Affiliations:** Institute of Cytology, Russian Academy of Sciences, St. Petersburg, 194064 Russian Federation

**Keywords:** tumor cells, cancer stem cells, autophagy, Sox2, Oct4, Autophinib

## Abstract

Numerous studies have already established that autophagy plays a central role in the survival of all cells, including malignant ones. Autophagy is a central cog in the general mechanism that provides the intracellular proteostasis determining cellular physiological and phenotypic characteristics. The accumulated data show that autophagy largely contributes to cancer cell stemness. Thus, autophagy modulation is considered one of the promising pharmacological targets in therapy aimed at cancer stem cell elimination. However, autophagy is a multi-stage intracellular process that involves numerous protein participants. In addition, the process can be activated simultaneously by various signaling modules. Therefore, it is no small feat to select an effective pharmacological drug against autophagy. What’s more, the search for potential chemotherapeutic agents that could eliminate cancer stem cells through pharmacological inhibition of autophagy is still under way. In the present work, we selected a panel of autophagy inhibitors (Autophinib, SBI-0206965, Siramesine, MRT68921, and IITZ-01), some of whom have been recently identified as effective autophagy inhibitors in cancer cells. Using A549 cancer cells, which express the core stem factors Oct4 and Sox2, we evaluated the effect of these drugs on the survival and preservation of the original properties of cancer stem cells. Among the agents selected, only Autophinib demonstrated a significant toxic effect on cancer stem cells. The obtained results demonstrate that autophagy inhibition by Autophinib downregulates the expression of the Sox2 protein in A549 cells, and that this downregulation correlates with a pronounced induction of apoptosis. Moreover, Autophinib-treated A549 cells are unable to form spheroids, which indicates a reduction in stemness. Thus, among the drugs studied, only Autophinib can be considered a potential agent against cancer stem cells.

## INTRODUCTION

Currently, autophagy is considered a promising molecular target for cancer cell therapy. Autophagy is known to play a crucial role at all stages of oncogenesis: i.e., during dissemination of cancer cells from the primary tumor, and, accordingly, during the formation of dissociated tumor cells, and during epithelial-mesenchymal transition, and, thus, during metastasis. In addition, it also maintains the cancer stem cell phenotype, thus, providing drug resistance and renewal of tumor. Considering that autophagy is the basis of the various phenotypic and physiological characteristics of cancer cells, in this work we studied the impact of autophagy inhibition on cancer cell elimination in vitro. Using literature data, we arrived at a panel of poorly studied pharmacological drugs that inhibit autophagy and can serve as anti-cancer agents. The pharmacological agent Autophinib, which was synthesized in 2017, seems to be a promising autophagy inhibitor (IC_50_ = 90 and 40 nM); it acts through the inhibition of lipid kinase Vps34 (IC_50_ = 19 nM in vitro) [1, 2]. Vps34 is involved in the formation of the pre-autophagosomal membrane, which serves as the basis for the production of autophagosomes, and is regulated by the kinases Ulk1 and Ulk2 [3]. Ulk1 and Ulk2 induce autophagy; therefore, inhibition of both Ulk1/2 and Vps34 blocks autophagy at its very early stage. We used two Ulk1/2 inhibitors in our study: SBI-0206965 (IC_50_ = 108 and 711 nM for Ulk1 and Ulk2, respectively) and MRT68921 (IC_50_ = 2.9 and 1.1 nM for Ulk1 and Ulk2, respectively). It was determined in 2015 that MRT68921 and SBI-0206965 are specific autophagy inhibitors; later, they were identified as potential anti-tumor agents [4, 5, 6, 7, 8]. In 2018, IITZ-01 was shown to act as an effective autophagy inhibitor [9]. IITZ-01 demonstrated high anti-tumor activity by inhibiting autophagy through lysosomal destabilization in in vitro and in vivo experiments in breast cancer models [9]. The drug Siramesine was first synthesized in 1995 as an anxiolytic, due to its ability to act as a selective sigma-2 receptor agonist, which recruits various psychotropic substances in the brain [10]. It is currently known that Siramesine effectively blocks autophagy through lysosomal destabilization in tumor cells [11]. Thus, Siramesine and IITZ-01 inhibit autophagy at late stages, when mature autophagosomes are unable to fuse with lysosomes due to disintegration of the latter for further degradation of the intercellular material. Cells with blocked autophagy are usually characterized by a high amount of autophagosomal structures accumulated in the cytoplasm.

Thus, according to numerous reports, the above autophagy inhibitors can effectively eliminate tumor cells both in vitro and in vivo. However, these agents have not been studied sufficiently enough to make a conclusion on the effectiveness of their use in cancer therapy. The ability of the selected pharmacological agents to effectively eliminate tumor cells was studied in A549 cancer cells, which demonstrate stemness. Cancer stem cells are very resistant to chemotherapy; hence, there is a good model system for in vitro screening for potential anti-tumor agents.

## EXPERIMENTAL

**Cell cultures**

A549 cancer cells were cultured in DMEM medium (Biolot, Russia) supplemented with 10% fetal bovine serum (FBS, Hyclone, USA) at 37°C and 5% CO_2_. Cells were obtained from the Center for Collective Usage "Vertebrate Cell Culture Collection". The following inhibitors were used in the study: Autophinib (5 μM), SBI-0206965 (1 μM), Siramesine (0.5 μM), MRT68921 (1 μM), and IITZ-01 (1 μM). All inhibitors were purchased from Selleckhem (USA).

**Cells transduction and analysis**

Lentiviral vector carrying the SORE6-mCherry reporter was kindly provided by Gordeev S.A. (Institute of Cytology of the Russian Academy of Sciences). A549 cells were transduced with the lentiviral vector using the protocol described in [12]. The fluorescence of mCherry was detected using a Becton Dickinson FACscan flow cytometer (USA) in the ECD-A channel.

**Spheroid formation**

A549 cells were cultured in hanging drops in non-adherent Sarstedt plates (Germany). Cells were pretreated with the indicated concentrations of autophagy inhibitors for three days. Cells were then detached from the plates using 1 : 1 trypsin–versene solution and seeded at a density of 4,000 cells per drop. Spheroid colonies of ≥ 50 μm were analyzed after seven days using an inverted TS100-F microscope (Nicon, Japan).

**Cell viability assay**

The number of viable and dead cells was assessed by flow cytometry. Cells were detached from the plates with 1 : 1 trypsin–versene solution and centrifuged. DAPI (1 μg/μl) was added to the suspension of viable cells; cells were incubated for 20 min at room temperature and analyzed on a Becton Dickinson FACscan flow cytometer (USA). The DAPI stain passes through the membrane of permeabilized cells, which makes it possible to identify dead cells.

## RT-PCR

Total RNA was isolated using TRIZOL reagent (Evrogen, Russia) according to the manufacturer’s protocol. Reverse transcription was performed using MMLV reverse transcriptase, 2.5 μg of RNA, and 1 μg of random hexaprimers based on the manufacturer’s instructions (Evrogen). Quantitative RT-PCR was carried out using real-time PCR kit from Evrogen containing SYBR Green on a 7500 Real-time PCR System (Applied Biosystems, USA). The following primers were used:

sox2 (F) – TTGCTGCCTCTTTAAGACTAGGA,

sox2 (R) – CTGGGGCTCAAACTTCTCTC;

gapdh (F) – GAGGTCAATGAAGGGGTCAT,

gapdh (R) – AGTCAACGGATTTGGTCGTA.

**In vitro analysis of Caspase-3 activity**

Cells were lysed in a buffer containing 50 mM HEPES (ICN, USA) pH 7.4, 0.1% CHAPS (Sigma), 0.5% IGEPAL-100 (ICN), and 5 mM DTT (Sigma) for 30 min at 4°C. An equal quantity of proteins from lysates was added to the reaction solution (40 mM HEPES pH 7.4, 0.1% CHAPS, 1 mmMM DTT, and 40 μM of AcDEVD-AMC fluorogenic substrate (Sigma)). The mixture was incubated for 1 h at 37°C. Fluorescence was measured using a GloMax®-Multi Jr detection system.

**Immunoblotting**

Cells were lysed in PBS containing 1% NP-40, 0.5% sodium deoxycholate, 0.1% SDS, protease, and phosphatase inhibitors and then centrifuged. Protein concentrations in samples were estimated using the Bradford assay; an equal amount of protein from each sample was loaded on the gel. Antibodies against Oct4, Sox2 (Santa Cruz, USA), LC3 (Cell Signaling, USA), and α-tubulin (Sigma) were used as primary antibodies. Anti-mouse rabbit antibodies and anti-rabbit goat antibodies conjugated to horseradish peroxidase were used as secondary antibodies. Proteins were detected by enhanced chemiluminescence (ECL, Amersham, United Kingdom). The obtained results were densitometry analyzed using ImageJ software. Values were normalized to the control load (α-tubulin) and expressed in relative units.

**Statistical data analysis**

The obtained data were analyzed using GraphPad Prism version 8 software package. The results are presented as a mean ± standard error of the mean. Mean values were compared using Student’s t-test with Bonferroni correction.

## RESULTS

**Autophinib exerts a pronounced cytotoxic effect on A549 cancer cells**

**Fig. 1:**
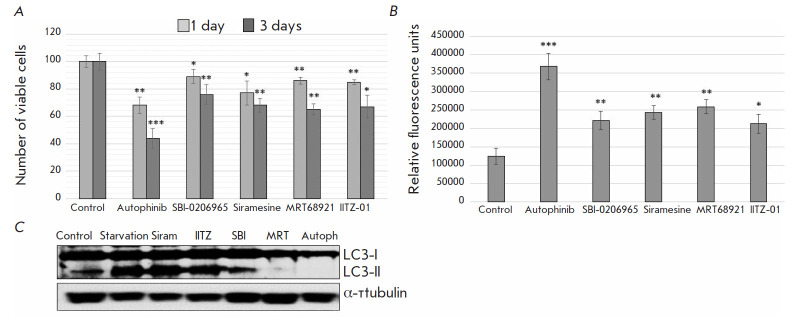
The effect of autophagy inhibitors on A549 cancer cell survival. (*A*) – Flow cytometry analysis of the number of living cells after treatment with Autophinib, SBI-0206965, Siramesine, MRT68921, and IITZ-01. Results are obtained after DAPI staining of the cell population. The cells were counted on days 1 and 3 of treatment with the indicated agents (n = 10,000 events). Error bars correspond to mean ± SEM (n = 3), * *p* < 0.05, ** *p * < 0.05, *** *p* < 0.005. (*B*) – *In vitro *caspase-3 assay in the control A549 cells and A549 cells treated with Autophinib, SBI-0206965, Siramesine, MRT68921, and IITZ-01 after 1 day. Error bars correspond to mean ± SEM (n = 3), * *p * < 0.05, ** *p * < 0.05, *** *p * < 0.005. (*C*) – Immunoblotting of cell lysates obtained from the control A549 cells and A549 cells under conditions of serum starvation for 4 h. The A549 cells were treated with Autophinib, SBI-0206965, Siramesine, MRT68921, and IITZ-01 for 4 h under conditions of serum starvation. The Anti-LC3-I/II and -α-tubulin antibodies were used

Working concentrations of the pharmacological agents Autophinib, SBI-0206965, Siramesine, MRT68921, and IITZ-01 were selected from published data based on the following principles: (1) the selected concentration does not cause death in > 50% of cells after 24 h, and (2) the selected concentration makes it possible to study stemness of surviving cells after 3 days of treatment. The obtained data showed that Autophinib (5 μM), SBI-0206965 (1 μM), Siramesine (0.5 μM), MRT68921 (1 μM), and IITZ-01 (1 μM) caused the death of ≤ 30% of A549 cells after one day of treatment (Fig. 1A). The number of dead A549 cells increased to 40% three days after treatment with SBI-0206965, Siramesine, MRT68921, and IITZ-01 (Fig. 1A). Autophagy inhibitor Autophinib exerted a pronounced cytotoxic effect on both day 1 (slightly > 30% of dead cells) and day 3 after treatment (~60% of dead cells) (Fig. 1A). Caspase-3 activity assay in vitro showed that all autophagy modulators above activate apoptosis in A549 cells, with Autophinib demonstrating the most pronounced pro-apoptotic effect (Fig. 1B). SBI-0206965, Siramesine, MRT68921, and IITZ-01 enhanced caspase-3 activation approximately twofold compared to the control 1 day after treatment; Autophinib increased caspase-3 activity approximately 3-fold (Fig. 1B). Thus, Autophinib showed the highest effectiveness in eliminating A549 cancer cells.

In order to assess the effectiveness of autophagy inhibition by the selected concentration range of the pharmacological agents Autophinib, SBI-0206965, Siramesine, MRT68921, and IITZ-01, we used anti-LC3 protein antibodies. The second form of this protein, LC3-II, is known to be formed through conjugation of the cytosol form of LC3 (LC3-I) with phosphatidylethanolamine on the surface of newly formed autophagosomes. Therefore, LC3-II is assumed to specifically label autophagosomes and autophagolysosomes and can be an indication of enhanced autophagy in cells. Based on the obtained results, it appears that the autophagic activity in A549 cells induced by 4-h serum starvation is effectively inhibited by Autophinib, SBI-0206965, and MRT68921 (Fig. 1C). Treatment with Siramesine and IITZ-01 resulted in LC3-II accumulation in cells similar to serum starvation in the absence of the abovementioned agents. This is apparently due to the fact that the mechanism of autophagy inhibition by Siramesine and IITZ-01 mainly has to do with lysosomal destabilization and, thus, does not interfere with autophagy at early stages until the accumulation of LC3-II-labeled autophagosomes. It is interesting to note that, of all the Ulk1/Ulk2 inhibitors used, MRT68921 showed the highest effectiveness in inhibiting autophagy in A549 cells compared to SBI-0206965 (Fig. 1C). Autophinib fully prevents LC3-II accumulation, which is an indication of effective suppression of this process in cells.

**Autophinib downregulates Sox2 expression in A549 cancer cells**

**Fig. 2:**
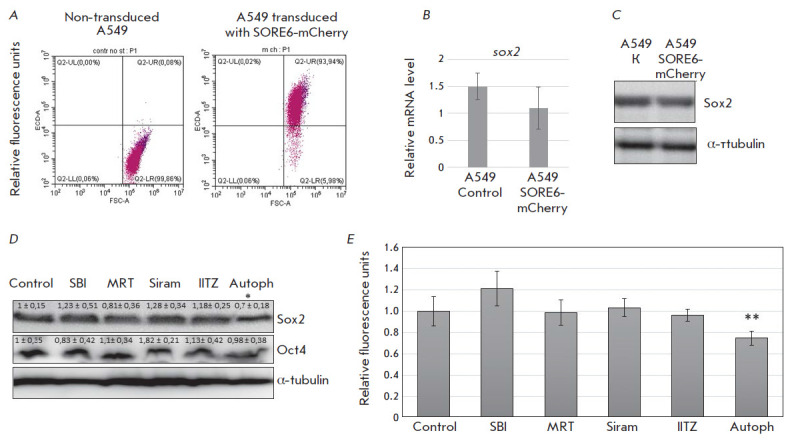
The effect of autophagy inhibitors on Sox2 and Oct4 expression in A549 cancer cells. (*A*) – Flow cytometry analysis of non-transduced A549 cells and cells transduced with the lentiviral SORE6-mCherry vector. Fluorescence was detected in the red (ECD-A) channel. (*B*) – Analysis of *sox2 *mRNA expression by quantitative RT-PCR in the control A549 cells and cells treated for 1 and 3 days with the indicated drugs. Expression was normalized to *gapdh*. (*C*) – Immunoblotting of cell lysates for the Sox2 protein in non-transduced A549 cells and cells transduced with the SORE6-mCherry vector. *α-tubulin *was used as a load control. (*D*) – Immunoblotting of cell lysates for the Sox2, Oct4, and α-tubulin proteins in A549 cells after treatment with the indicated drugs for three days. The results are densitometric and presented as mean ± SEM (n = 3), * *p * < 0.05. (*E*) – Flow cytometry analysis of fluorescent emission of the reporter vector SORE6-mCherry in A549 cells after treatment with the indicated drugs for three days. Flow cytometry results are presented as diagrams. The mean fluorescence value was determined based on three independent experiments, excluding cell autofluorescence. Error bars correspond to mean ± SEM (n = 3), ** *p * < 0.05

Transcription factors with robust expression in embryonic stem cells are considered stemness drivers in cancer cells. Moreover, Sox2 and Oct4 expression levels correlate with the histological degree of tumor malignancy; these proteins are often used as prognostic markers of cancer cell response to therapy and disease outcome [13, 14]. In order to fully characterize the Sox2 and Oct4 expression levels in A549 cancer cells, the cells were transduced with a lentiviral vector carrying the SORE6-mCherry fluorescent reporter [12]. SORE6-mCherry contains six repeats of promoter region sequences for binding Sox2 and Oct4. Recruitment of these transcription factors results in the induction of transcription of the red fluorescent protein mCherry. This fluorescent protein has been designed for the detection of cancer stem cells [12]. Figure 2A shows that transduced A549 cells have a significant fluorescence intensity in the red spectrum, which allows for the identification of the Oct4 and Sox2 activities. According to the obtained results, lentiviral transduction does not change the Sox2 expression at neither the gene nor protein level, which indicates the applicability of the model used in this study (Fig. 2 B and C). We further studied the effect of Autophinib, SBI-0206965, Siramesine, MRT68921, and IITZ-01 on the Oct4 and Sox2 protein levels in A549 cells 3 days after treatment. The Oct4 protein level was shown to remain the same after treatment with each of the pharmacological agents, while the Sox2 level decreased in A549 cells after treatment with Autophinib and did not change in the presence of SBI-0206965, Siramesine, MRT68921, or IITZ-01 (Fig. 2D). Analysis of the Oct4 and Sox2 expression levels in A549 cells transduced with the SORE6-mCherry lentiviral vector confirmed a decrease in stemness in cancer cells in the presence of Autophinib (Fig. 2E). Transformed A549 cells were cultured in the presence of the autophagy inhibitors Autophinib, SBI-0206965, Siramesine, MRT68921, and IITZ-01 for 3 days and then analyzed using flow cytometry. Based on the obtained data, the fluorescence intensity of the reporter vector SORE6-mCherry decreases significantly in A549 cells after treatment with Autophinib (Fig. 2E). Western blotting results (Fig. 2D) allow us to assume that the decrease in fluorescence intensity is associated with a reduction in Sox2, but not Oct4, activity: the level of the latter remains the same in the presence of Autophinib. We can assume that the cytotoxic effect of Autophinib is accompanied by a decrease in cell stemness and, therefore, has a pronounced effect. Thus, the obtained data demonstrate that, of the selected panel of autophagy inhibitors, only Autophinib altered the stemness characteristics of A549 cells.

**Treatment of A549 cells with Autophinib inhibits the formation of tumor spheroids**

**Fig. 3:**
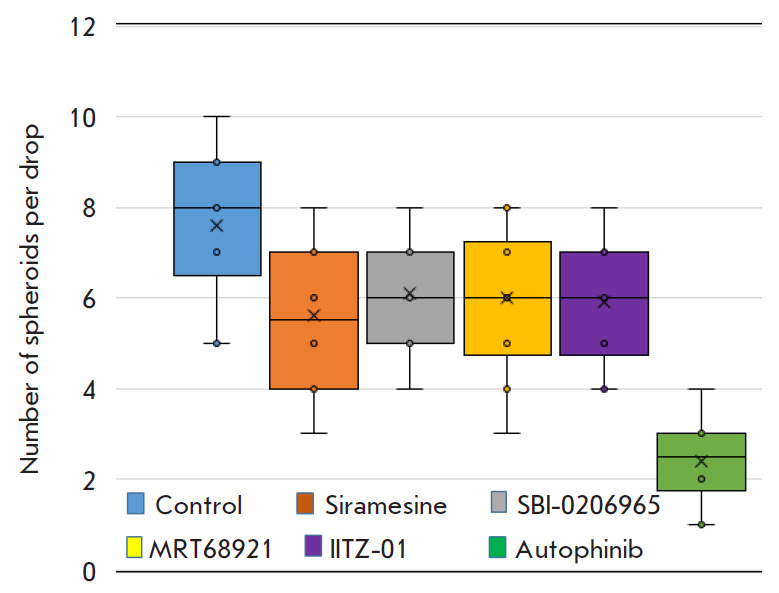
Effect of autophagy inhibitors on the ability of A549 cells to form spheroids. The results are presented as box plots. Spheres with a size of at least 50 μm were counted in one drop

Tumor spheroids are three-dimensional (3D) structures formed by cancer cells that imitate solid tumors in vivo in numerous key aspects such as heterogeneous architecture, internal gradients of signaling factors, nutrients, and oxygenation. Tumor spheroids are a more adequate model of drug resistance compared to monolayer cultures [15]. To evaluate the malignant potential of the surviving A549 cells after treatment with Autophinib, SBI-0206965, Siramesine, MRT68921, and IITZ-01, we analyzed the ability of cancer cells to form spheroids. A549 cells were pre-cultured in the presence of the abovementioned agents for 3 days, dissociated, and cultured in special conditions for the formation of 3D structures. The obtained data shows that A549 cells treated with SBI-0206965, Siramesine, MRT68921, and IITZ-01 could form spheroids of ≥ 50 μm, although to a lesser extent compared to the control (Fig. 3). Apparently, the reduced number of spheroids formed from the pretreated A549 cells is due to the apoptotic program initiated by these agents, which had been preserved in the cells used for the formation of the 3D structures. Autophinib can significantly block the potential of cancer cells to form spheroids, which may be due to severe impairment of intracellular proteostasis in A549. Apparently, Autophinib can have an irreversible impact on cancer cells, leading to their inability to restore homeostasis and resulting in their elimination.

## DISCUSSION

Recently, numerous autophagy-inhibiting compounds have been developed. Their use is hoped to result in the massive death of cancer cells, accompanied by low toxicity for healthy human cells [16, 17]. Some autophagy-modulating drugs are already utilized in clinical practice (rapamycin, chloroquine, and hydroxychloroquine), while others are undergoing clinical trials (mTOR kinase inhibitors) [17, 18]. The fact that around 70% of clinical studies are now focused on the role of autophagy in oncogenesis points to the high expectations vested in the use of autophagy modulation in the treatment of cancer [19].

This study was an attempt to assess the potential therapeutic significance of Autophinib, SBI-0206965, Siramesine, MRT68921, and IITZ-01 in the elimination of A549 cancer stem cells. We showed that only Autophinib – among all the examined autophagy inhibitors – exerts a pronounced antitumor effect by decreasing cancer cell stemness, inducing apoptosis in tumor, and preventing cancer cell population renewal. Apparently, the antitumor effects of Autophinib are achieved through severe disruption of the cellular proteostasis caused by the inhibition of Vps34, followed by the inhibition of not only autophagy. This is because lipid kinase Vps34 is one of the main producers of phosphatidylinositol-3-phosphate in the cell, which, in turn, recruits the corresponding proteins to the membranes. Thus, Vps34 plays a key role not only in autophagy induction and formation of the primary membrane with recruitment of membrane protein complexes, but also in endocytosis [20, 21]. For this reason, Vps34 inhibition leads to the suppression of membrane vesicle formation, which is necessary in both autophagy and endocytosis, since it disrupts intracellular homeostasis. In addition, endocytosis mostly mediates the interaction between cells in the tumor, and its impairment can separate cancer cells [22]. Apparently, inability of A549 cells to form spheroids after they are treated with Autophinib is also due to the damage caused to intercellular communication. Thus, the antitumor effect of Autophinib is associated with not only autophagy inhibition, but also the functioning of other signaling pathways. For this reason, pharmacological agents such as SBI-0206965 and MRT68921 that block autophagy through a targeted inhibition of proteins Ulk1 and Ulk2 turn out to be less toxic to A549 cancer stem cells than Autophinib. It is interesting to note that Siramesine and IITZ-01 also demonstrated a poor tumor elimination activity in A549 cells, despite the fact that their action is associated with a destabilization of lysosomes, which are required in both autophagy and endocytic pathways. Nevertheless, inhibition of kinase Vps34 has turned out to be a less effective strategy in eliminating cancer stem cells in both our study and other works. Vps34 activity is shown to be necessary for the expansion of cancer stem cells in the liver: RNA-interference of this protein has the opposite effect in the form of tumor growth suppression in vivo [23]. In addition, pharmacological inhibition of Vps34 effectively eliminates the cancer stem cell population in the liver and also inhibits tumor growth in vivo [23]. Inhibition of Vps34 activity effectively eliminates cancer stem cells in the presence of combination therapy in a model of tumor spheroids [24]. A combination therapy with 5-fluorouracil and the drug 36-077, which is a Vps34 inhibitor, mainly kills tumor cells with the stem cell phenotype [24].

The results obtained and data published by us indicate that a pharmacological approach to autophagy inhibition in cancer cells should be aimed at cross-signaling pathways. Monotherapy based on autophagy inhibition is currently considered ineffective [19]. The main reasons for this are the following: (1) the dual role of autophagy in cancer, (2) the absence of therapeutically suitable autophagy inhibitors, and (3) the lack of knowledge about cross-interactions between autophagy and other signaling pathways in the cell. Co-inhibition of autophagy and endocytic pathways through Vps34 inhibition can be a good strategy for eliminating cancer stem cells. Therefore, the study of autophagy in terms of vesicular transport can be considered a promising research path.

## Abbreviations

Vps34: phosphatidylinositol 3-kinase catalytic subunit type 3
